# A Systematic Review of the Adequacy of Undergraduate Ophthalmology Education in the United Kingdom

**DOI:** 10.7759/cureus.94186

**Published:** 2025-10-09

**Authors:** Sajad Hussain, Rohan Aziz, Riccardo Cheloni, Mohammed Mohyudin

**Affiliations:** 1 Ophthalmology, Mid Yorkshire Trust, Wakefield, GBR; 2 Ophthalmology, Calderdale and Huddersfield NHS Trust, Huddersfield, GBR; 3 Optometry, Bradford University, Bradford, GBR

**Keywords:** ai in medical education, curriculum management, general ophthalmology, systematic review, teaching feedback

## Abstract

Ophthalmology is a vital yet often underemphasized part of undergraduate medical training in the United Kingdom (UK). Despite the high occurrence of eye-related presentations in clinical settings, evidence shows variation in teaching exposure, content, and assessment across medical schools. This systematic review, registered with the International Prospective Register of Systematic Reviews (PROSPERO)(registration number: CRD42025110189), examined undergraduate ophthalmology education in the UK. Comprehensive searches were carried out in Medline, Cumulative Index to Nursing and Allied Health Literature (CINAHL), Web of Science, Excerpta Medica database (Embase), and the Cochrane Library up to May 2025. Eligible studies, published from 2000 onwards, evaluated ophthalmology teaching at the undergraduate level. Data extraction and thematic analysis were independently performed by two reviewers, with disagreements resolved by a third. Quality assessment used the Joanna Briggs Institute (JBI) Critical Appraisal Checklist, Cochrane Risk of Bias 2 (RoB 2), and Non-randomized Studies of Interventions (ROBINS-I) tool, depending on study design. Seven studies met the inclusion criteria, including national surveys, interventional trials, and observational research. Ten major themes emerged: limited curriculum time, curriculum variability, low student confidence in fundoscopy, misdiagnosis in primary care, positive responses to e-learning and blended learning, equipment and supervision limitations, assessment-driven engagement, low curriculum priority, and lack of national guidance. Evidence showed significant variation in teaching duration (three to 15 days), low self-reported competence, and the benefits of blended and e-learning methods for engagement and satisfaction. Undergraduate ophthalmology education in the UK remains fragmented and inconsistent in priority. Standardising curricula, mandating minimum placement durations, incorporating ophthalmology into summative assessments, and expanding blended learning could improve competence and ensure graduates meet essential ophthalmic care standards.

## Introduction and background

It has been reported that ophthalmic conditions represent a significant proportion of consultations in the United Kingdom (UK) general practice and emergency departments. Specifically, Rawlings et al. [1] estimated that between 5% and 19% of consultations in general practice were related to the eyes. Similarly, the authors reported that approximately 4% of emergency department consultations were for ophthalmic issues. With an ageing population and the increasing prevalence of age-related eye disease, these figures are expected to rise.

Despite this large volume of medical consultations being conducted by non-ophthalmology specialists, it has been reported that undergraduate medical education does not dedicate sufficient time to ophthalmology training within the university curriculum [2,3]. It has been found that junior doctors and general practitioners report low confidence in diagnosing and treating ophthalmic conditions [2,3]. This can result in patients’ safety issues due to the potential for life- and sight-threatening nature of conditions with ocular symptoms.

Curricular restructuring has contributed to this shortfall. The General Medical Council’s Outcomes for Graduates (2018) defined broad learning expectations but omitted speciality-specific competencies, prompting many medical schools to reduce teaching in smaller disciplines such as ophthalmology [3]. In response, the International Council of Ophthalmology (ICO) and the Royal College of Ophthalmologists (RCOphth) have issued guidelines outlining essential knowledge and skills, including visual acuity assessment, pupillary reflex testing, ocular motility, and fundoscopy [4-5]. However, the extent to which these competencies are delivered and assessed across UK medical schools remains unclear.

This systematic review evaluates the adequacy of undergraduate ophthalmology education in the UK by comparing current teaching provision and learning outcomes with national and international curricular standards, aiming to identify deficiencies and guide future curriculum development.

## Review

Methods

This systematic review was conducted in accordance with the Preferred Reporting Items for Systematic Reviews and Meta-Analyses (PRISMA) guidelines. The review protocol was registered with the International Prospective Register of Systematic Reviews (PROSPERO) (registration number: CRD42025110189). 

Search strategy

A systematic search was performed in Medical Literature Analysis and Retrieval System Online (MEDLINE), Cumulative Index to Nursing and Allied Health Literature (CINAHL), Web of Science, Excerpta Medica database (Embase), and the Cochrane Library, using the search terms “Ophthalmology”, “Undergraduate Medical Education”, “Curriculum”, “Medical Students”, and “United Kingdom” or “UK”. Searches covered up to May 2025.

Eligible studies were those evaluating undergraduate ophthalmology teaching in the UK. All study designs were considered, including quantitative, qualitative, and mixed-methods research (e.g., cross-sectional surveys, randomised controlled trials of teaching methods, cohort studies, and curriculum evaluations). In addition, grey literature such as national reports, medical school surveys, and conference abstracts was included if it contained original data. Only studies published from 2000 onwards were eligible to reflect changes in medical curricula following the Tomorrow’s Doctors reforms.

The exclusion criteria specified that studies would be excluded if they were conducted outside the UK, focused on postgraduate training or continuing professional development, or were not peer-reviewed articles.

Data extraction and analysis

Two reviewers (SH and RA) independently reviewed the titles and abstracts to assess if they met the inclusion criteria. The two reviewers (SH and RA) then independently examined the full texts of the selected articles to determine if they met the inclusion criteria. Again, disagreements between the two reviewers (SH and RA) were resolved by the third reviewer (MM). 

Given the heterogeneity of study designs and outcomes, a qualitative thematic analysis was performed on the full texts, which met the inclusion criteria. A three-stage process was used to integrate findings from the included studies. Two reviewers (SH and RA) independently extracted and coded the relevant segments line by line. Codes with similar meanings were combined into a broader descriptive theme. This approach allowed synthesis of survey data, interventional trials, reviews, and editorials into a coherent narrative, highlighting areas of convergence and divergence across the literature. Discrepancies in coding and theme development were resolved through discussion or arbitration by a third reviewer (MM). The kappa coefficient of inter-reviewer reliability was 0.90. 

Quality assessment of studies

The quality assessment of studies was performed using several critical appraisal tools. Due to the included studies varying in design, design-specific critical appraisal tools were used to assess the quality of studies. Cross-sectional surveys and the retrospective audit were appraised with the Joanna Briggs Institute (JBI) Critical Appraisal Checklist [6]. The JBI checklist was used due to its suitability for questionnaire-based and prevalence studies. The randomised controlled trial was assessed with the Cochrane Risk of Bias 2 (RoB 2) tool [7]. The prospective non-randomised study was evaluated with the risk of bias in Non-randomized Studies of Interventions (ROBINS-I) tool, which addresses confounding and participant selection in experimental designs (Table 1) [8]. 

**Table 1:** Quality assessment of articles JBI: Joanna Briggs Institute; RoB 2: Risk of Bias 2; ROBINS-I: Non-randomized Studies of Interventions; CEQ: course experience questionnaire; MCQ: multiple choice question

Author and Year	Appraisal tool	Selection / Sampling	Measurement Validity	Confounding	Missing Data	Analysis/Reporting	Overall Risk of Bias
Statham et al., 2008 [3]	JBI Criteria	Low (consecutive hospital cases)	Some concern (retrospective records, subjective adverse outcome)	High (no adjustment)	Low	Low	Moderate
Baylis et al., 2011 [9]	JBI Criteria	Low (national survey, 83% response)	Some concern (self-reported single respondent)	Not applicable (descriptive)	Low	Low	Low–Moderate
Hill et al., 2017 [10]	JBI Criteria	Low (93% response rate)	Some concern (self-reported)	Not applicable	Low	Low	Low–Moderate
Welch et al., 2011 [11]	JBI Criteria	Some concern (56% response, brief methods)	Some concern	Not applicable	Low	Low	Moderate–High
Schulz et al., 2014 [12]	JBI Criteria	Some concern (43% Year 4, 33% final-year response)	Some concern (self-reported confidence)	Some concern	Low	Low	Moderate
Petrarca et al., 2018 [13]	Cochrane RoB 2	Low (topic randomisation)	Low (standardised exam & survey)	Some concern (topics randomised, not individuals)	Low	Low	Some concerns
Doyle et al., 2024 [14]	ROBINS-I	Moderate (defined cohorts but voluntary survey)	Low–Moderate (validated CEQ, MCQ)	Serious (different academic years, confounding)	Serious (low response rates)	Low	Serious

Results

Study Characteristics

**Figure 1:**
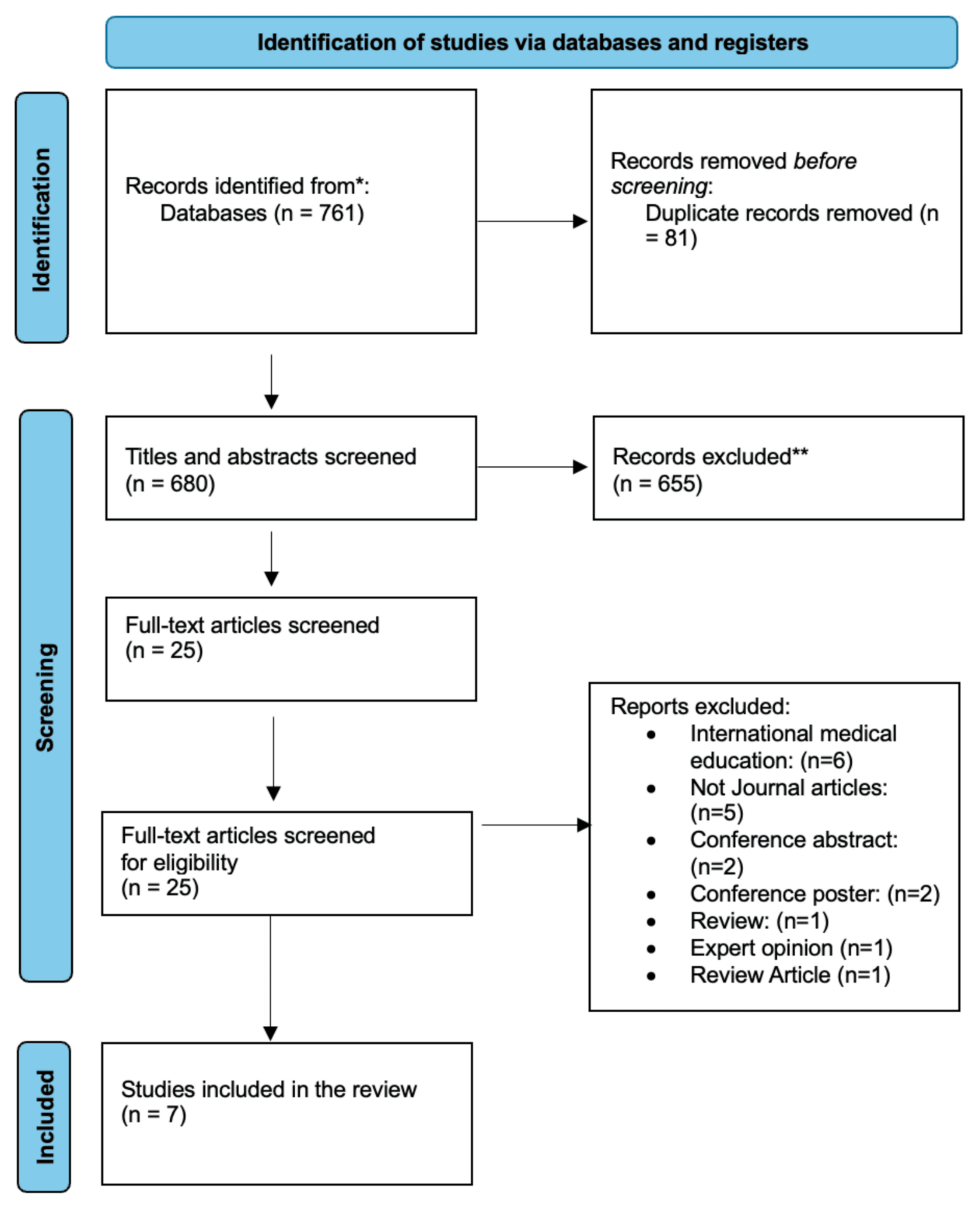
A PRISMA flowchart outlining the study selection process PRISMA: Preferred Reporting Items for Systematic Reviews and Meta-Analyses

Seven hundred and sixty-one titles met the criteria; once duplicates were removed, 680 remained, which were retrieved. Title and abstract screening identified 25 articles, and after full-text screening, only seven articles were included in the review. Of the seven articles, four were UK national survey studies [9-12], two were interventional studies [13-14], and one was an observational study [3]. Collectively, they examine curriculum duration and variability, student competence, educational innovations, and recommendations for improvement (Figure 1, Table 2).

**Table 2:** Study charateristics RCT: randomized controlled trial

Study	Design	Population/Sample	Focus/Intervention	Key Outcomes
Statham et al. [3]	Observational (diagnostic accuracy)	Primary health care providers	Misdiagnosis of acute eye disease	Diagnostic accuracy, common errors
Baylis et al. [9]	Survey (cross-sectional)	UK medical schools	Ophthalmology education provision	Teaching hours, curriculum content
Hill et al. [10]	Survey (cross-sectional)	29 UK medical schools	Undergraduate ophthalmology curriculum (present & future)	Teaching time, attitudes, and proposed reforms
Welch et al. [11]	Survey (cross-sectional)	25 UK medical schools	Ophthalmology teaching delivery	Curriculum trends, comparisons
Schulz et al. [12]	Cross-sectional	Medical students & trainees	Confidence in fundoscopy skills	Factors associated with diagnostic confidence
Petrarca et al. [13]	RCT	Undergraduate medical students	eLearning vs traditional teaching	Knowledge outcomes, learning effectiveness
Doyle et al. [14]	RCT Non-randomised	Medical students	Blended ophthalmology teaching	Student learning gains (knowledge & skills)

From the seven included studies, 10 common broader themes were derived. 

Limited curriculum time: Multiple studies report that ophthalmology receives very few teaching hours or is offered only as an elective. Baylis et al. [9] reported a median of around one week of teaching with a reported range of three to seven days, whilst Hill et al. [10] reported some schools had no formal placement.

Curricular variability: Marked differences in ophthalmology teaching structure and requirements among UK medical schools. Welch et al. [11] found wide heterogeneity in required hours and attachments, with few including structured clinics, ward rounds, or surgery observations.

Low student confidence in fundoscopy: Students were lacking confidence in performing fundoscopy and interpreting findings. Schulz et al. [12] found that only 25% felt confident after training, and only 43% of final year students were confident in examining a dilated eye, and less than 40% were confident in identifying key fundus pathology. 

Misdiagnosis in primary care: Primary health care providers misdiagnose acute eye diseases due to knowledge gaps. Statham et al. [3] reported frequent misdiagnosis of acute red eye.

Positive response to eLearning: Students valued and showed improved outcomes with eLearning modules. Petrarca et al. [13] found significant knowledge gain in randomised controlled trials.

**Figure 2:**
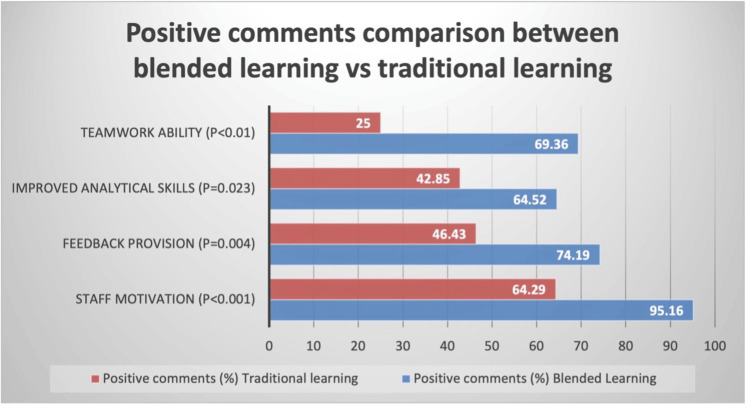
Doyle et al.'s survey results Reference: [14]

Positive response to blended learning: Doyle et al. [14] found that students showed improved performance and satisfaction when online and in-person teaching were combined (Figure 2). Petrarca et al. [13] found that e-learning helped improve exam performance compared with traditional lectures, with an improved mean examination score (Table 3). Doyle et al. [14] found higher exam scores in combined teaching vs. traditional teaching.

**Table 3:** Teaching data from Petrarca et al. Reference: [13]

Study	Teaching Type	Mean Score (%)	95% Confidence Interval ()	P-value
Petrarca et al. [13]	E-learning	58	55.7 – 59.6	p<0.05
	Traditional learning	55	53.1 – 56.8	

Equipment and supervision constraints: A lack of ophthalmoscopes, simulation devices, and supervisory availability limits practice opportunities. Hill et al. [10] reported limited access to specialist clinics.

Assessment drives engagement: When ophthalmology is assessed formally, students are more likely to prioritise learning. Baylis et al. [9] reported that medical schools with Objective Structured Clinical Examinations (OSCEs) stations report better engagement. Only 11 schools had formal summative ophthalmology assessments. 

Low priority in curriculum: Considered a minor speciality, the perception of ophthalmology as a low-priority or niche subject can reduce motivation. Hill et al. [10] reported that student comments reflect the belief that ophthalmology is not essential for finals.

National guidance and core competencies: Consensus that all graduates must master specific skills: visual acuity testing, red flag recognition, and basic eye examination [9-11]. Several studies highlighted a lack of standardisation and suggested that compliance with competency frameworks was often superficial [9,10].

Discussion

This systematic review identifies ongoing gaps and recent innovations in undergraduate ophthalmology education within the UK. Evidence consistently demonstrates that ophthalmology has not maintained parity with other core components of the medical curriculum, resulting in reduced teaching time and inconsistent educational delivery across institutions. Across 10 major themes, the findings demonstrate a pattern of curricular compression, heterogeneity in structure, and observable implications for clinical competence. Following the revisions to the Tomorrow’s Doctors framework, ophthalmology was not specified as a core requirement, which contributed to reduced curricular inclusion across medical schools [15].

Although eye-related complaints account for a notable share of cases seen in both primary and emergency care, ophthalmology teaching occupies only a small proportion of undergraduate medical education [3,13]. Surveys of UK medical schools report wide variation in exposure, averaging 7.6 days (range 3.5-15 days), with some institutions offering no compulsory placement [9]. Similar findings were reported by Hill et al. [10], who attributed this variation to competition for curricular time and the absence of ophthalmology from national guidance documents. The reduction of structured ophthalmology exposure mirrors international trends and may limit the ability of new graduates to perform basic ophthalmic assessments [16].

In addition to limited duration, the content and delivery of ophthalmology education vary substantially among UK medical schools. Welch et al. [11] reported that only four of 18 responding schools taught all 13 of the recommended formal topics, and none covered all 10 recommended clinical skills [11]. Hill et al. [10] similarly documented broad variation in organisation, assessment, and educational environment across institutions, with no consistent national model. This heterogeneity contributes to inconsistent student outcomes and variable preparedness for clinical practice.

Empirical evidence also indicated that limited teaching time correlates with low self-reported confidence in ophthalmic skills. Schulz et al. [12] found that only 43% of final-year medical students were confident examining a dilated eye, and fewer than 40% felt confident identifying key pathologies such as papilledema or diabetic retinopathy. Confidence was significantly associated with greater opportunities for practice and structured feedback, suggesting that educational exposure directly influences self-efficacy. These findings are consistent with reports of diagnostic inaccuracy among general practitioners and emergency clinicians, where correct identification rates for common ophthalmic conditions ranged from 36% to 48% [3].

Several studies have explored the relationship between curricular emphasis and student motivation. Baylis et al. [9] reported that medical schools incorporating ophthalmology into summative OSCEs demonstrated higher engagement. Conversely, Hill et al. [10] observed that students perceived ophthalmology as non-essential for final examinations, which reduced independent learning motivation. These findings support the principle that assessment drives learning and suggest that inclusion of ophthalmology within summative frameworks may enhance student engagement and competence.

Emerging evidence supports the use of technology-enhanced teaching modalities to augment traditional instruction. In a randomised controlled crossover study, Petrarca et al. [13] found that e-learning modules produced slightly higher mean examination scores, but with no statistical significance between the scores. Petrarca et al. [13] found greater student satisfaction with e-learning compared with traditional lectures. Students valued flexibility and interactivity, indicating that e-learning can improve engagement without increasing contact time. Similarly, Doyle et al. [14] evaluated a blended-learning model integrating online flipped-classroom sessions with in-person seminars and patient-based teaching, reporting significantly greater satisfaction, enhanced analytical and teamwork skills, and improved feedback provision compared with an exclusively online format. Although examination performance did not differ significantly between groups, qualitative outcomes indicated strong student preference for blended delivery. Both studies emphasised that online learning should supplement, rather than replace, clinical experience.

Traditional lecture-based approaches remain prevalent, but studies demonstrate that interactive and blended models improve learner satisfaction and self-perceived competence [13,14]. However, Hill et al. [10] noted that access to ophthalmoscopes, simulation equipment, and supervised clinical practice remains limited in several institutions, potentially constraining the translation of theoretical knowledge into practical skill.

Innovative strategies have been proposed to address curricular constraints. Chada and Gooding [17] advocate embedding ophthalmology teaching through simulations, interprofessional sessions, alignment with licensing examinations, and modern tools such as smartphone fundoscopy, alongside national standardisation of competencies. Studies consistently call for nationally defined minimum standards encompassing visual acuity testing, recognition of red-flag symptoms, and competence in direct ophthalmoscopy [9,11].

Overall, the reviewed literature supports several convergent recommendations: implementation of a standardised national curriculum guided by the RCOphth and the ICO; integration of ophthalmology into summative assessments to enhance learning motivation; and adoption of blended, technology-assisted teaching models to optimise limited curricular time. While quantitative improvements in examination performance remain modest, these interventions demonstrably enhance learner satisfaction, engagement, and self-confidence. The collective evidence indicates that restoration of minimum educational standards, supported by modern delivery methods, is essential to sustain ophthalmology’s relevance within undergraduate medical education.

Evidence gathered from the studies indicates that more adjustments could be made to help standardise the curriculum to provide an adequate level of education for doctors to be competent in managing ocular conditions. Based on the studies, the following are areas that can be examined and potentially improved to improve the quality of ophthalmology education: Develop a national undergraduate ophthalmology curriculum for all medical schools to follow; Develop a minimum duration of ophthalmology placements across all medical schools; Develop multifaceted teaching methods incorporating eLearning tools, simulations and online classrooms to supplement lectures; Mandate practical skills such as fundoscopy, fluorescein staining, and lid eversion into summative assessments; Ensure the curriculum covers basics such as visual acuity testing, pupil testing, colour vision testing, lid eversion, fluorescein staining, fundoscopy and basic slit lamp examination; Ensure the curriculum is aligned with the ICO and RCOphth competency frameworks.

Limitations

The available evidence is limited by heterogeneity among the included studies, which makes direct comparison challenging. Much of the data are derived from low-quality evidence, with most data coming from self-reported survey-based research, raising concerns about response and selection bias, as well as poor correlation with actual competence. Reported outcomes tend to focus on knowledge, confidence, and satisfaction rather than long-term clinical performance or patient outcomes. Positive findings from e-learning and blended learning may also be influenced by publication bias. Furthermore, issues such as resource availability and supervision constraints are highly context-dependent. Moreover, there is a lack of recent longitudinal data to provide insights into trends over time. To address these gaps, further national audits and well-designed longitudinal studies are needed to more accurately track progress and guide future improvements. More randomised controlled studies are needed, as the ones available have design flaws. 

## Conclusions

Undergraduate ophthalmology education in the UK remains variable in duration, content, and assessment, despite the high prevalence of ophthalmic presentations in clinical practice. Several national surveys show significant differences in teaching hours and a continued lack of ophthalmology in core medical curricula. This fragmentation has been linked to low student confidence in key diagnostic skills, including fundoscopy, and limited clinical competence upon graduation. Recent advances in educational design, especially the use of e-learning and blended-learning models, offer promising strategies to increase ophthalmic exposure within crowded curricula. Randomised and comparative studies indicate that these approaches improve student satisfaction and perceived competence without requiring extra contact hours. Nonetheless, reliable access to equipment, supervision, and clinical placements remains crucial to turn theoretical improvements into practical skills. A national, standardised ophthalmology curriculum aligned with the guidelines of the RCOphth and the ICO could address current disparities and ensure all medical graduates meet a minimum competency standard. Incorporating ophthalmology into summative assessments, combined with modern blended-learning methods, could emphasise its educational importance and better prepare future clinicians to manage ocular conditions safely and effectively.

## References

[1] Rawlings A, Hobby AE, Ryan B (2024). The burden of acute eye conditions on different healthcare providers: a retrospective population-based study. Br J Gen Pract.

[2] Scantling-Birch Y, Naveed H, Tollemache N, Gounder P, Rajak S (2022). Is undergraduate ophthalmology teaching in the United Kingdom still fit for purpose?. Eye (Lond).

[3] Statham MO, Sharma A, Pane AR (2008). Misdiagnosis of acute eye diseases by primary health care providers: incidence and implications. Med J Aust.

[4] International Task Force on Opthalmic Education of Medical Students; International Council of Opthalmology (2006). Principles and guidelines of a curriculum for ophthalmic education of medical students. Klin Monbl Augenheilkd.

[5] (2025). The Royal College of Ophthalmologists. https://www.rcophth.ac.uk/training/undergraduate-ophthalmology.

[6] JBI JBI (2025). Critical appraisal tools. https://jbi.global/critical-appraisal-tools.

[7] Cochrane Methods (2025). Risk of Bias 2 (RoB 2) tool. https://methods.cochrane.org/risk-bias-2.

[8] Sterne JA, Hernán MA, Reeves BC (2016). ROBINS-I: a tool for assessing risk of bias in non-randomised studies of interventions. BMJ.

[9] Baylis O, Murray PI, Dayan M (2011). Undergraduate ophthalmology education - a survey of UK medical schools. Med Teach.

[10] Hill S, Dennick R, Amoaku W (2017). Present and future of the undergraduate ophthalmology curriculum: a survey of UK medical schools. Int J Med Educ.

[11] Welch S, Eckstein M (2011). Ophthalmology teaching in medical schools: a survey in the UK. Br J Ophthalmol.

[12] Schulz C, Hodgkins P (2014). Factors associated with confidence in fundoscopy. Clin Teach.

[13] Petrarca CA, Warner J, Simpson A (2018). Evaluation of eLearning for the teaching of undergraduate ophthalmology: a randomised controlled crossover study. Eye.

[14] Doyle AJ, Murphy CC, Boland F, Pawlikowska T, Ní Gabhann-Dromgoole J (2024). Education in focus: significant improvements in student learning and satisfaction with ophthalmology teaching delivered using a blended learning approach. PLoS One.

[15] (2025). Outcomes for graduates, plus supplementary guidance. https://www.gmc-uk.org/education/standards-guidance-and-curricula/standards-and-outcomes/outcomes-for-graduates.

[16] Spencer SK, Ireland PA, Braden J (2024). A systematic review of ophthalmology education in medical schools: the global decline. Ophthalmology.

[17] Chadha N, Gooding H (2021). Twelve tips for teaching ophthalmology in the undergraduate curriculum. Med Teach.

